# The Critical Role of Protein Arginine Methyltransferase *prmt8* in Zebrafish Embryonic and Neural Development Is Non-Redundant with Its Paralogue *prmt1*

**DOI:** 10.1371/journal.pone.0055221

**Published:** 2013-03-12

**Authors:** Yu-ling Lin, Yun-Jung Tsai, Yu-Fang Liu, Yi-Chuan Cheng, Chuan-Mao Hung, Yi-Jen Lee, Huichin Pan, Chuan Li

**Affiliations:** 1 Department of Biomedical Sciences, Chung Shan Medical University, Taichung, Taiwan; 2 Department of Medical Research, Chung Shan Medical University Hospital, Taichung, Taiwan; 3 Department of Biochemistry and Molecular Biology, Chang Gung University, Kwei-Shan, Tao-Yuan, Taiwan; Peking University, China

## Abstract

Protein arginine methyltransferase (PRMT) 1 is the most conserved and widely distributed PRMT in eukaryotes. PRMT8 is a vertebrate-restricted paralogue of PRMT1 with an extra N-terminal sequence and brain-specific expression. We use zebrafish (*Danio rerio*) as a vertebrate model to study PRMT8 function and putative redundancy with PRMT1. The transcripts of zebrafish *prmt8* were specifically expressed in adult zebrafish brain and ubiquitously expressed from zygotic to early segmentation stage before the neuronal development. Whole-mount *in situ* hybridization revealed ubiquitous *prmt8* expression pattern during early embryonic stages, similar to that of *prmt1.* Knockdown of *prmt8* with antisense morpholino oligonucleotide phenocopied *prmt1-*knockdown, with convergence/extension defects at gastrulation. Other abnormalities observed later include short body axis, curled tails, small and malformed brain and eyes. Catalytically inactive *prmt8* failed to complement the morphants, indicating the importance of methyltransferase activity. Full-length *prmt8* but not *prmt1* cRNA can rescue the phenotypic changes. Nevertheless, cRNA encoding Prmt1 fused with the N-terminus of Prmt8 can rescue the *prmt8* morphants. In contrast, N-terminus- deleted but not full-length *prmt8* cRNA can rescue the *prmt1* morphants as efficiently as *prmt1* cRNA. Abnormal brain morphologies illustrated with brain markers and loss of fluorescent neurons in a transgenic fish upon *prmt8* knockdown confirm the critical roles of *prmt8* in neural development. In summery, our study is the first report showing the expression and function of *prmt8* in early zebrafish embryogenesis. Our results indicate that *prmt8* may play important roles non-overlapping with *prmt1* in embryonic and neural development depending on its specific N-terminus.

## Introduction

Posttranslational modifications (PTMs) provide fine switches for regulating the structure and function of proteins. Protein arginine methylation is one type of PTM that mostly occurs on arginine residues embedded in arginine and glycine rich motifs in various nucleic acid binding proteins [1], [2]. Protein arginine methyltransferases (PRMTs) can catalyze the transfer of methyl groups from *S*-adenosylmethionine (AdoMet) to specific arginine residues in these proteins. According to their ability to catalyze the formation of asymmetric or symmetric dimethylarginines (ADMA or SDMA), the vertebrate PRMTs can be divided as type I or type II. The type I PRMT includes PRMT1, PRMT2, PRMT3, PRMT4 (CARM1), PRMT6 and PRMT8. The only well-documented type II enzyme is PRMT5 [1], [2]. PRMT7, though was classified as type II, has been reported as a type III enzyme that catalyzes the formation of monomethylarginine [3].

PRMT1 is the first identified [4] and the most broadly distributed type I PRMT [2]. It is also the most conserved PRMT with sequence similarity higher than 90% in vertebrates and higher than 70% between human and budding yeast [2]. It is involved in various cellular processes including signal transduction modulation and transcriptional regulation [5], [6]. For example, interaction of PRMT1 with the cytoplasmic domain of interferon (IFN) α receptor [7] indicate its role in INF signaling. On the other hand, PRMT1 is a coactivator for some nuclear receptors as well as various transcription factors such as p53 and YY1 [5], [6]. Specifically, asymmetric dimethylation of histone H4 Arg3 (H4R3me2a) by PRMT1 is part of the histone code. PRMT1, together with other PRMTs including PRMT2, 4, 5, 6, and 7 are co-activators/repressors for epigenetic regulation by histone arginine methylation [2].

PRMT8, first named as HRMT1L3, shares 80% sequence identity with PRMT1 and is encoded by a gene at human chromosome 12p13 [8]. This gene was identified in a screen of genes specifically expressed in neural precursor cells from *Sox1-gfp* knock-in mice [9]. Not only the amino acid sequence but also the intron-exon organization are conserved between PRMT1 and PRMT8 in vertebrates [10]. PRMT8 can be identified in mammals and fish but not in an invertebrate chordate *Ciona intestinalis*, thus appears to be a vertebrate-specific PRMT1 paralogue [10]. Type I activity of PRMT8 was demonstrated later [11] and PRMT8 has similar substrate preference as PRMT1 [12].

PRMT8 is the only PRMT with tissue-restricted expression pattern. Brain-specific expression pattern was first shown by Northern blotting [11]. Neuron-specific somatosensory distribution was further demonstrated by *in situ* hybridization [13]. Because a myristoylation motif is around the predicted initiation methionine of human PRMT8, removal of the initiating methionine followed by myristoylation of the newly exposed N-terminal glycine was suggested. Plasma membrane localization and myristoylation of transfected GFP-PRMT8 were shown in HeLa cells [11]. The results may implicate a putative upstream role of PRMT8 in certain signaling pathways. However, dominant nuclear localization of PRMT8 was observed in mouse central nervous neurons by a PRMT8-specific antibody [14], arguing against the plasma membrane localization of PRMT8. Initiation from the third AUG and no myristoylation of PRMT8 were proposed [14]. In this way, the possibility that PRMT8, like PRMT1 and most other PRMTs, participates in epigenetic histone modification increases.

PRMT8 can form homodimers as well as heterodimers with its highly conserved paralogue PRMT1 [11]. PRMT1 and PRMT8 share high sequence identity and the major difference is that PRMT8 has an extra N-terminal sequence for about 76 amino acid in human. Deletion of the N-terminal 60 amino acids increased the methyltransferase activity of PRMT8 *in vitro*, indicating that the region might be involved in regulating the enzyme activity [15].

High sequence identity as well as conserved substrate preference of PRMT8 and PRMT1 indicate similar catalytic activity and implicate putative redundancy. However, PRMT8 is the only mammalian PRMT with tissue specificity. The neuron-specific expression of PRMT8 as well as its restricted distribution in vertebrates may suggest a novel function of PRMT8 in the vertebrate nervous system. As we had analyzed the embryonic expression and function PRMT1 in zebrafish [16] and PRMT8 is highly conserved in vertebrates [10], we determined to use zebrafish as a vertebrate model to study PRMT8. We analyzed the early embryonic expression pattern of Prmt8 and observed *prmt8*-knockdown phenotypes in zebrafish. We performed rescue experiments with *prmt*8 or *prmt1* cRNAs to the *prmt*8 or *prmt1* AMO-injected embryos (morphants) to investigate the function of Prmt8, and the putative redundancy of Prmt1 and Prmt8.

## Materials and Methods

### Zebrafish rearing

Adult zebrafish were maintained on a 14-h light/10-h dark cycle at 28°C. All embryos were collected by natural spawning and staged according to Kimmel et al. [17]. The animal use protocol has been reviewed and approved by the Institutional Animal Care and Use Committee of Chung Shan Medical University. The wildtype AB strain and *Tg(huC::eGFP)* fish were obtained from the Taiwan Zebrafish Core Facility (TZCF) and Taiwan Zebrafish Core Facility at Academia Sinica (ZCAS).

### Bioinformatic analyses of zebrafish Prmt8

To predict the coding sequence of zebrafish Prmt8, human (NP_062828) and mouse PRMT8 amino acid sequences (Q6PAK3) were used as the homologues and the zebrafish genome (BX784029) was retrieved from NCBI database for genomescan. The predicted result was then modified according to its homology with *Fugu* PRMT8 amino acid sequence (BK004166). Four zebrafish EST sequences (AW077391, EB915765, EB929481, and EB929248) were retrieved from database for the assembly of zebrafish Prmt8. The deduced Prmt8 sequence is consistent with the protein arginine N-methyltransferase 8-B sequence in NCBI (Q5RGQ2.2). The human and zebrafish genomic DNA segments containing the *prmt8* genes were analyzed by UCSC Genome browser.

### Zebrafish *prmt8* constructs

Adult zebrafish brain tissue was homogenized and total RNA was extracted with Tri-reagent (Molecular Research Center, Inc., Cincinnati, OH, USA). To obtain the complete coding sequence of zebrafish Prmt8, a primer set (ZF8-NF3: 5′-ATGGGACTGAGGCACTCAT-3′; and ZF8R2: 5′-GGTGAAGTCTAAGTCACGCTCA) was used in RT-PCR to amplify adult fish brain cDNA. The coding region was further subcloned into pGEX-4T to express glutathione S-transferase (GST)-fused Prmt8.

For evaluating the efficiency of the MOs, the sequence coding the N-terminus of Prmt8 was amplified by PCR with the primer set ZF8-BglII-F (5′-GAAGATCTATGGGACTGAGGCAC-3′) and ZF8N-KpnI-R (5′-ATGGTACCTGTCATCTCCTCTGG-3′), and the *BglII-KpnI* restriction fragment was subcloned into the pEGFP-N3 vector to result in pEGFPN3-Prmt8 N1-96.

To prepare zebrafish *prmt8*-specific probe, the sequence coding the N-terminal region (containing 289 nt) that shares the least homology with Prmt1 was amplified by ZF8-NF3 and ZF8-R289 (5′-CAACCCAGAGGAGATGAC-3). The fragment was cloned into a pGEM-T Easy vector and the pGEM-prmt8-289 plasmid was used for riboprobe preparation.

### mRNA expression analyses by RT-PCR

Total RNA was isolated from embryos at different stages of embryogenesis and different adult tissues by TRIzol reagent (Molecular Research Center, Inc.). First strand cDNA was synthesized from 3 µg of total RNA by SuperScript III Reverse Transcriptase (Invitrogen). RT-PCR was performed with the primer set ZF8-NF2 (5′-TCCCAGTCCCTACAACCATC-3′) and ZF8-NR2 (5′-AAGGTGGAGGAGACGGAACT-3). Amplification of the elongation factor 1α with the primer set Ef-1 (5^′^-GCTCAAGGAGAAGATCG-3^′^ ) and Ef-2 (5^′^-TCAAGCATTATCCAGTCC-3^′^ ) was used as an internal control. Amplified cDNA products were resolved by agarose electrophoresis and visualized by ethidium bromide staining.

### Whole-mount *in situ* hybridization

Embryos were collected, allowed to develop at 28.5°C until the appropriate stage, dechorionated by pronase treatment, and then fixed by incubation overnight in 4% paraformaldehyde at 4°C. After fixation, embryos were dehydrated and stored at -20°C in 100% methanol. *In situ* hybridization was performed according to Westerfield [18]. Basically after rehydration, proteinase treatment, and prehybridization, hybridization was performed with 100 – 200 ng of digoxigenin-UTP labeled riboprobes. For the preparation of the *prmt8* antisense RNA probe, the pGEM-prmt8-289 plasmid was linearized by *EcoRI* restriction enzyme and the RNA was transcribed with SP6 RNA polymerase. For the preparation of the sense RNA probe, the plasmid was cleaved by *SalI* restriction enzyme and the RNA was transcribed with T7 RNA polymerase. The embryos were washed and incubated with anti-DIG antiserum and then stained. Embryos are then mounted in 100% glycerol for observation using a dissecting microscope (ZEISS AXioskop2; CarlZeiss, Oberkochen, Germany). Plasmids for preparing probes of *tbx6, sox17, myoD, ntl, pax2.1* and *otx2* were obtained as generous gifts from Dr. Ching-Hua Hu (Department of Life Sciences, National Taiwan Ocean University) and Dr. Bon-Chu Chung (Institute of Molecular Biology, Academia Sinica, Taiwan).

### Knockdown of Prmt8 by antisense morpholino oligonucleotide and rescue cRNA injection

Two antisense morpholino oligonucleotides of *prmt8* (*prmt8* MO1: 5′- ACCGCGATGAGTGCCTCAGTCCCAT -3′; MO2: 5′- TGCTCTCCGCCTCCGCCATCTTTCT -3′) against translational start sites and 5- mispair MO2 (5′-TGgTgTCCGgCTCCcCCATgTTTCT; lowercase letters indicate the mutated bases) for specificity control were designed and purchased from Gene Tools (Philomath, OR, USA). Control embryos were injected with a non-specific standard morpholino oligonucleotide (5-CCTCTTACCTCAGTTACAATTTAT-3′) or 5-base mismatch MO2. We also co-injected 1.5 fold (w/w) p53 AMO (5′-GACCTCCTCTCCACTAAACTACGAT-3′) with the *prmt8* AMO as suggested by Robu et al. [19] to avoid apoptosis induced through the off-target activation of *p53*. However, as co-injection with or without the p53 AMO revealed similar phenotypes and death/ defect rates (Fig. S1), we did not include p53 AMO in later experiments. AMO was injected into 1-cell embryos with a microinjector Nanojector II (DrummondScientific Company, Broomall, PA, USA). The full *prmt8* coding region was cloned into the vector pCS2^+^. Rescue experiments were performed with *prmt8* cRNA synthesized *in vitro* using the mMESSAGEmMACHINE kit according to the manufacturer’s protocol (Ambion Europe Ltd. Huntingdon, UK). The G2A and N-terminal deletion mutations were introduced by site directed mutagenesis to produce the mutant by the following primer set: forward primer ZF8-G2A-F (5′-CGGGATCCATGGCACTGAGGCACT-3′) or ZF8-Ndel-F (5′-CGGGATCCATGGCCAAAACTGCTC-3′) and reverse primer ZF8-R (5′-GCTCTAGATTACCGCATTTTGTAGTC-3′). The PCR products were cleaved by *BamHI* and *XbaI* and subcloned into the pCS2^+^ vector. The zebrafish Prmt8 N-terminal 94 amino acid coding region was amplified by PCR with the primer set ZF8-NF3 (5′-ATGGGACTGAGGCACTCATC-3′) /ZF8-XhoI R (5′-TCTCGAGCTCCTCTGGGTTG-3′) and the fragment was subcloned into a TA vector. The zebrafish Prmt1 coding region was amplified with the primer set ZF1-XhoI F (5′-CCTCGAGATGACGTCCAAAG-3′) /ZF1-XbaI R (5′-CTCTAGACTAGCGCATTCTG-3′) and the *XhoI - XbaI* restriction fragment was subcloned behind Prmt8N94 in the TA vector. The *EcoRI* and *XbaI* restriction fragment containing the Prmt8N94-Prmt1 coding sequence was subcloned into pCS2^+^. Catalytic inactive Prmt8 with SGT to AAA mutations at the conserved AdoMet-binding site as Balint et al. [20] were introduced by site directed mutagenesis to produce the catalytic mutant. To observe embryos from the *Tg(huC::eGFP)* fish, images were taken by Leica DM2500 epifluorescence microscope with DFC490 CCD camera.

### Zebrafish embryonic extract preparation, western blot analyses

Zebrafish embryos of different developmental stages were manually deyolked as described by Link et al. [21] and dissolved in extraction buffer (150 mM NaCl, 100 mM Tris-HCl, pH 7.5, 5% glycerol, 1 mM DTT, 1% triton X-100, 1 mM PMSF and complete protease inhibitor cocktail from Roche) and then homogenized (400 µl/100 embryos) by a homogenizer (IKA T10; IKA Works; Staufen, Germany). The homogenate was further centrifuged at 17530 x g at 4°C for 20 min. The supernatant was stored at –20°C as the embryonic extract. Aliquots of the zebrafish embryonic extract (30 µg) were resolved by SDS-PAGE followed by western blot analyses. Anti-methylarginine antibodies ASYM24 (Upstate Biotechnology, Lake Placid, NY, USA) were diluted according to the suggestions of the manufacturer.

### In vitro methylation

Expression of recombinant GST-Prmt8 fusion protein from pGEX-Prmt8 transformed *E. coli* DH5α cells was induced by 0.1 mM of IPTG at 37°C for 3.5 hr. The cells were lysed by sonication in lysis buffer (150 mM NaCl, 10 mM Tris-HCL pH 8.0, 5 % glycerol, 1 mM EDTA, 1 mM PMSF, 1 % TritonX-100, 1 mM dithiothreitol and 2 mg/ml lysozyme). The GST-Prmt8 fusion protein in the supernatant of the *E. coli* extract was purified with glutathion agarose as suggested by the manufacturer (GE Healthcare). Recombinant mouse fibrillarin with the six-histidine tag was expressed and purified as described [22]. *In vitro* methylation was conducted as described previously [23].

## Results

### Characterization of the zebrafish *prmt8* sequence

We deduced the DNA sequence encoding zebrafish Prmt8 by assembling the genomic as well as EST sequences. The *prmt8* gene is located on chromosome 4 with conserved gene structure as *prmt1*. The encoded polypeptide is 85 amino acids longer than the one previously reported in NCBI (NP_001038507) and is consistent with the protein arginine N-methyltransferase 8-B sequence in NCBI (Q5RGQ2.2). There is another gene on chromosome 18 designated as zebrafish protein arginine methyltransferase 8 in Ensembl (ENSDARP00000107466 or GI:326675987) with the same exon-intron organization. However, no EST data could support its expression. We also could not detect its expression by RT-PCR (data not shown). It is thus likely to be a non-expressed pseudogene.

**Figure 1:**
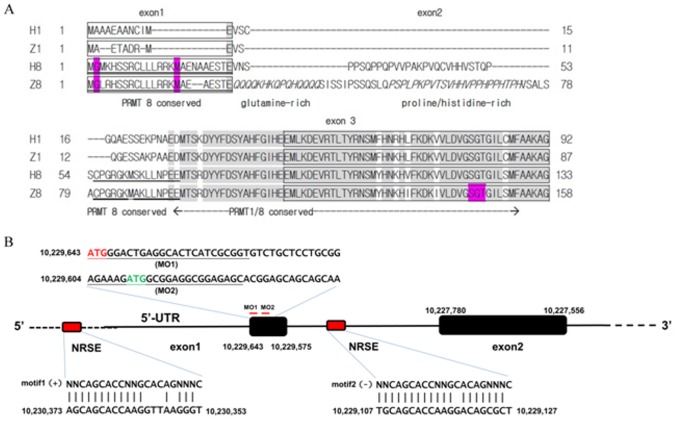
N-terminal amino acid sequence and critical regions in the 5’-region of zebrafish *prmt8*. (A) Comparison of the N-terminal amino acid sequences of human and zebrafish Prmt1 and Prmt8. The N-terminal amino acid sequences of human PRMT1 (H1; NP_938074.2), zebrafish Prmt1 (Z1; NP_956944.2), human PRMT8 (H8; NP_956944.2) and zebrafish Prmt8 (Z8; Q5RGQ2.2) were aligned by COBALT (http://www.ncbi.nlm.nih.gov/tools/cobalt/). The amino acids encoded by different exons are marked by boxes. The grey shading shows identical amino acid sequence in the four entries. The conserved sequence in human and zebrafish PRMT8 before the PRMT1/PRMT8 conserved region are underlined. The glutamine-rich and proline/histidine-rich sequences are shown in italics. The second glycyl residue that might be myristoylated, the second methionine residue that corresponds to the third and alternative initiating methionine proposed in rat PRMT8 [14], and the SGT at the conserved AdoMet-binding site that were mutated to AAA to make catalytic-inactive methyltransferase are highlighted. (**B**) Schematic representation of part of the zebrafish *prmt8* gene encompassing the 5’-flanking region, exon I to II including intron I. The accession number for zebrafish *prmt8* gene is Q5RGQ2.2. The region displayed comprises genome coordinates of the zebrafish chromosome 4 (in Jul. 2010 Zv9/danRer7 assembly). The potential NRSEs encompassed 21 nucleotides (10,230,373-10,230,353, and 10,229,127-10,229,107 complementary strand) were aligned by the consensus NRSE motif derived from the “core” motif (5′-NNCAGCACCNNGCACAGNNNC-3′). The red boxes show the NRSEs located within the 5′-flanking and the first intron of the *prmt8* gene. The MO1 and the MO2 binding site to pair with the *prmt8* initiating ATG and the second ATG are indicated.

The N-terminal methionine of zebrafish Prmt8 is followed by a glycine residue as that of human or mouse PRMT8 (Figure 1A). Excluding the extra N-terminus, PRMT8 is highly conserved with PRMT1 starting from the MTSK/(R)DYY sequence. The N-termini of zebrafish and human PRMT8 share high sequence similarity in the 25 amino acid residues encoded by exon 1. Another conserved region is the 15 amino acid residues before the conserved PRMT1/PRMT8 sequences (Figure 1A). The sequence in between is diverse among the mammalian and fish PRMT8. This region contains the proline-rich domain in human PRMT8 that has been shown to interact with the SH-3 domain [15]. In zebrafish, a unique glutamine-rich sequence is present before a proline and histidine-rich region in this region (Figure 1A).

By *in silico* analyses using genome browser, neuron-restrictive silencing elements (NRSEs) /repressor element 1 (RE-1) were identified in both the 5’-flanking region and intron 1 of human *prmt8.* Similar NRSE organization was then identified in the zebrafish *prmt8* gene (Figure S2; Figure 1B). The NRSEs are recognized by neuron-restrictive silencing factor /RE-1 silencing transcription factor (NRSF/REST), a master negative regulator of neurogenesis that represses neuronal genes in non-neuronal tissues and was proposed to be a master regulator of neuronal gene expression [24]. The NRSE / RE-1 sequences further explain the neuron specificity of the *prmt8* gene.

### Spatial and temporal expression of *prmt8* in embryonic development

**Figure 2:**
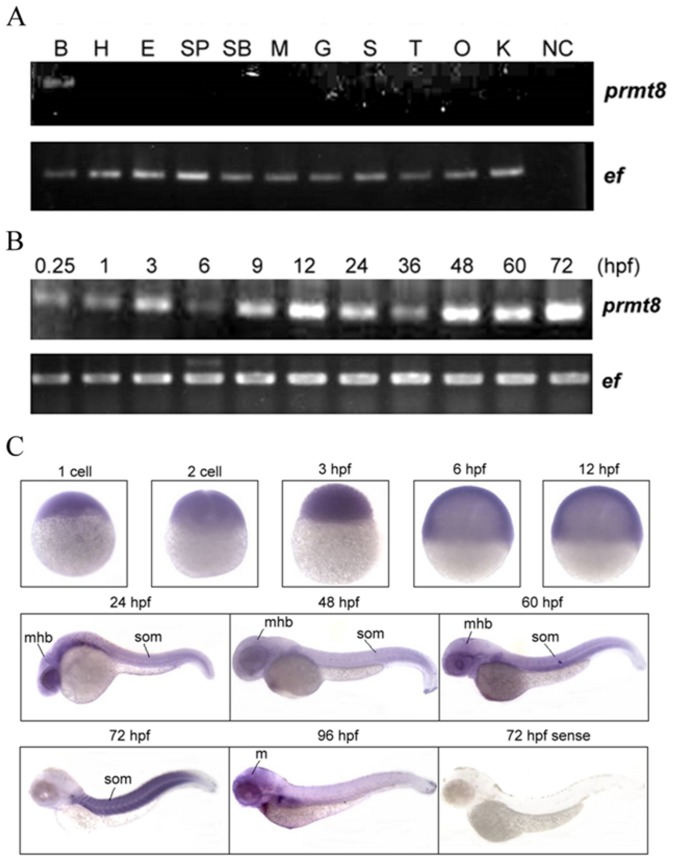
Expression analysis of *prmt8* RNA in zebrafish adult tissues and embryos. (A) Expression of *prmt8* in different adult tissues was analyzed by RT-PCR. EF indicates the RT-PCR product of elongation factor. (B: brain; SP: spleen; G: gill; O: ovary; H: heart; SB: swim bladder; S: skin; E: eye; M: muscle; T: testis) (B) RT-PCR of *prmt8* RNA prepared from 0.25 hpf to 72 hpf embryos. (C) The spatial and temporal expression of *prmt8* RNA from 1 cell to 96 hpf by WISH in zebrafish embryonic development. (m: midbrain; Mhb: mid-hindbrain boundary; som: somites).

Analyses of the RT-PCR product of zebrafish brain RNA confirmed the zebrafish *prmt8* sequence and revealed no alternative splicing. As expected, the message was expressed in brain but not in any other adult tissues we examined (Figure 2A).

We then analyzed *prmt8* expression during early developmental stages in zebrafish. The message of *prmt8* was detected from 0.25 hours post fertilization (hpf) throughout 72 hpf by RT-PCR (Figure 2B). We further investigated the expression pattern by whole-mount *in situ* hybridization (WISH). We had demonstrated that *prmt1*, the highly conserved paralogue of *prmt8*, was ubiquitously expressed and is critical for zebrafish early development [16]. To eliminate false-positive detection of the highly conserved *prmt1*, we designed a probe hybridizing to the 5’-region of *prmt8,* which corresponds to the specific N-terminus of Prmt8 and shares least sequence similarity with *prmt1*. Ubiquitous *prmt8* expression was detected at early developmental stages from one cell to 12 hpf (Figure 2C). The expression of *prmt8* in the eyes and brain, especially at the mid-hindbrain boundary and the hindbrain was clearly detected at 24 hpf. The expression in the head regions continued to 60 hpf. Even distribution in the somites was also detected during these periods. The *prmt8* signals were restricted to the somites at 72 hpf. The signals faded in the somites and were present mainly in the midbrain at 96 hpf (Figure 2C).

### Zebrafish *prmt8* knocked-down embryos showed early developmental defects

The putative N-terminal sequence of zebrafish Prmt8 has an initiating methionine followed by a glycine residue as those in mouse and human [11]. We first designed a translation-blocking MO against this 5’-most AUG (MO1). However, Kousaka *et al* suggested a putative initiation site at the third AUG in mammalian *prmt8* [14]. We thus designed another MO (MO2) corresponding to this AUG (second AUG in zebrafish *prmt8*) (Figure 1B).

**Figure 3:**
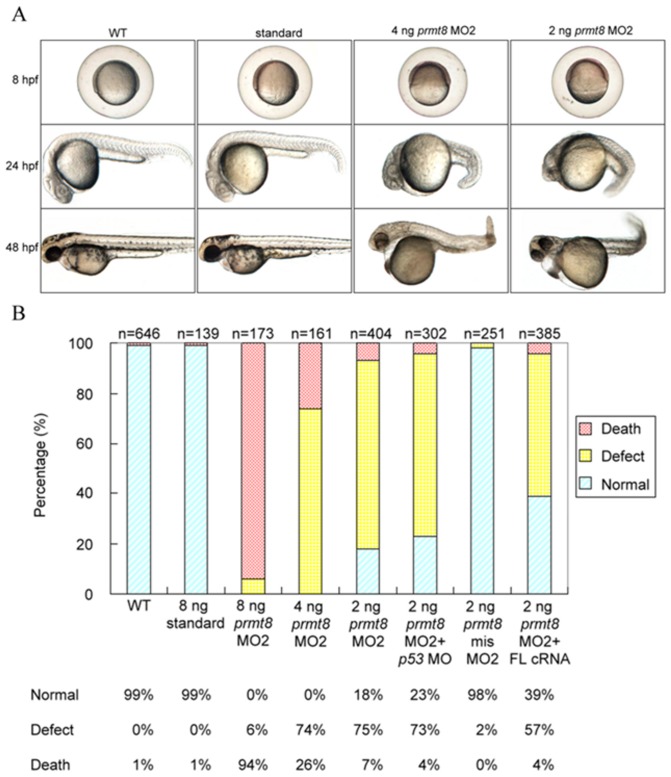
Phenotypes of zebrafish Prmt8 morphants. (A) Phenotypes of zebrafish embryos not injected (WT; wildtype), injected with control AMO (standard), or injected with 2 or 4 ng *prmt8* MO2 at 8, 24, and 48 hpf. (B) Percentage of normal, dead, or defective zebrafish embryos not injected, injected with standard AMO, *prmt8* MO2 (2, 4 and 8 ng), 2 ng of MO2 co-injected with *p53* MO, 5- mispair MO2, or 2 ng of MO2 co-injected with full-length *prmt8* cRNA. The embryos were examined at 24 hpf. Embryos with any of the gross phenotypic changes including curved tails, small and malformed brain and eyes, short yolk stalk, enlarged and swollen yolk and cardiac edema were classified as “defect”.

The overall morphologies of the MO-injected embryos (morphants) were evaluated. Injection of MO1 at high dose (8 ng) resulted in abnormal phenotypes and defect rates close to that of the MO2-injected embryos at a lower dose (2 ng) (Figure 3A). Increased MO2 leads to high death rates as 85% of the 8 ng MO2-injected morphants were dead at 24 hpf. The death rate (24 hpf) reduced to 50% and less than 5% when 4 ng and 2 ng of MO2 were injected respectively (Figure 3B). We thus used 2 ng of MO2 for most of the subsequent knock-down experiments to allow the high portion of viable yet defective morphants. Defects as delayed epiboly and thicker germ ring in the MO2 morphants could be clearly observed as early as 8 hpf (Figure 3A). The gross phenotypic changes of the *prmt8* morphants observed at 24 and 48 hpf include curved tails, small and malformed brain and eyes, short yolk stalk, enlarged and swollen yolk and cardiac edema (Figure 3A). The death and defective rate of the morphants were dose-dependent (Figure 3B), suggesting the specificity of the MOs. Injection of the 5-base mismatch MO2 and the standard control MO showed insignificant defect and death rate (Figure 3B), further supporting the specificity of the knockdown.

To further evaluate the specific blocking effects, the *prmt8* MO was co-injected with a reporter plasmid expressing the N-terminus of Prmt8 fused with EGFP. Embryos injected with the plasmid alone showed strong green fluorescence. Co-injection of the fusion plasmid and *prmt8* MO2 significantly reduced the fluorescent signals in the embryos, suggesting the suppressive effects of the MO (Figure S3).

To further confirm that the phenotypes of the *prmt8* morphants are specific due to the knockdown, we rescued the morphants with *prmt8* cRNA. Co-injection of full-length *prmt8* cRNA with MO2 led to increased ratios with normal phenotypes (Figure 3B). The results suggest that the early embryonic expression of *prmt8* is critical to zebrafish embryogenesis.

### Marker analyses for defective early embryogenesis in the *prmt8* morphants

**Figure 4:**
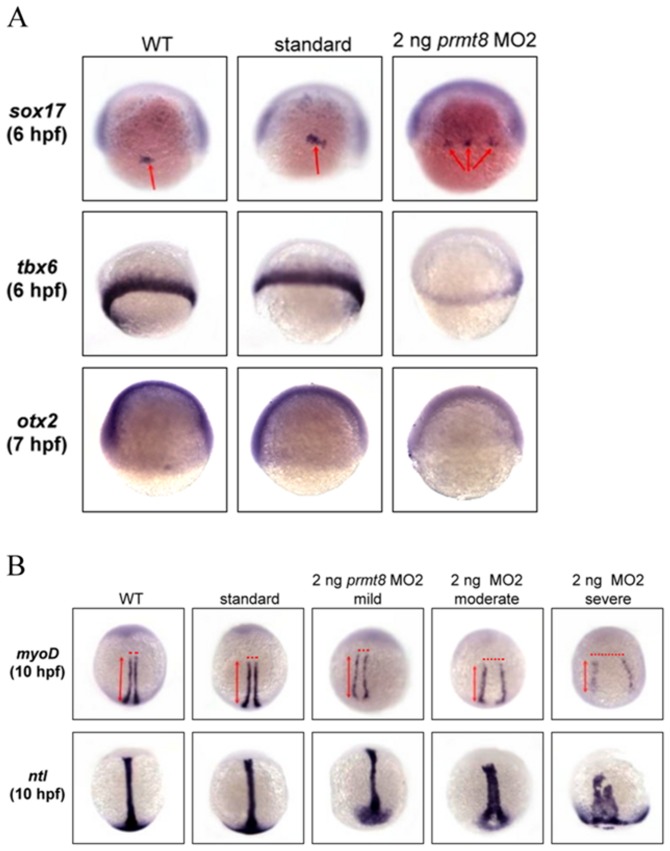
Defective phenotypes at gastrulation and early segmentation stage of the zebrafish *prmt8* morphants. (A) Zebrafish embryos not injected (WT), injected with control AMO (standard) or 2 ng of *prmt8* MO2 were stained with an endodermal marker *sox 17* (animal pole views) or a mesodermal marker *tbx6* (side views) at 6 hpf, and an ectodermal marker *otx2* at 7 hpf. (B) Zebrafish embryos not injected, injected with control AMO or 2 ng of *prmt8* MO2 were stained with *myoD* or *ntl* at 11 hpf (dorsal views).

Although early embryonic Prmt8 expression had not been reported in mammals, we showed that zebrafish *prmt8* was ubiquitously expressed before the segmentation stage and knockdown of *prmt8* led to severe phenotypes in early embryonic stages. We thus further analyzed the *prmt8* morphants with early genetic markers to characterize the defective phenotypes for the critical function of *prmt8* during early embryogenesis. As delayed epiboly and thicker germ ring were observed in the MO2 morphants at 8 hpf (Figure 3A), endodermal and mesodermal markers were used to monitor cell-type specific expression and migration. An endodermal marker *sox17* expressed in endodermal precursor cells and dorsal forerunner cell is critical for endoderm formation. In the *prmt8* morphants, the *sox17*-stained forerunner cells remained as a few separate spots and did not converge well at around 50% epiboly (the beginning of gastrulation at 6 hpf; Figure 4A). The mesodermal marker *tbx6* is a member of T-box transcription family and a posterior ventral mesoderm marker at gastrulation. Even though the localizations of *tbx6* appeared to be similar in control and morphant embryos, reduced *tbx6* expression level was detected in the *prmt8* morphants (Figure 4A). We also use another marker *otx2* to follow the early ectodermal development. Similar distribution but reduced expression of *otx2* was detected in the *prmt8* morphants (Figure 4A). These results indicate *prmt8* knockdown resulted in abnormal gene expression or cell distribution in all three layers.

Since body axis shortening and distortion were observed in the morphants, we also analyzed the morphants with markers related to somite formation. Analysis with a muscle and somite-specific marker *myoD* at early segmentation stage (11 hpf) revealed that the width between the bilateral adaxial cells increased, while the length of the body-axis decreased in the morphants. The width was broadened more at the posterior than the anterior end (Figure 4B). Staining with notail (*ntl,* expressed in the ring mesoderm and endodermal precursors around the margin as a pan-mesendodermal marker) also showed shortened but widened notochords in the *prmt8* morphants. The axial mesendoderm failed to migrate to the anterior. In the most severe ones, the axial cells did not converge (Figure 4B).

### Type I activity of zebrafish Prmt8 is critical for early embryogenesis

As PRMT8 is a type I protein arginine methyltransferase that can catalyze the formation of asymmetric dimethylarginines, we prepared recombinant GST-Prmt8 protein to examine its activity. The enzyme catalyzes the methylation of a typical type I substrate fibrillarin (Figure S4A), showing that zebrafish Prmt8 is catalytically similar to human PRMT8.

**Figure 5:**
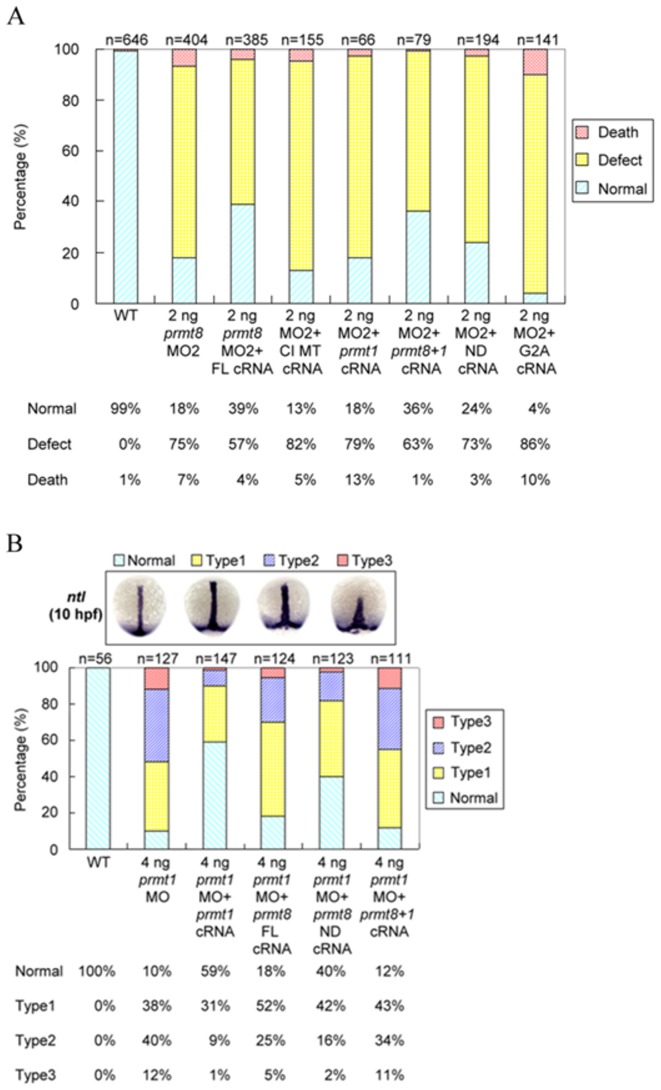
Rescue of *prmt8* or *prmt1* morphants with *prmt8* or *prmt1* cRNA. (A) Frequencies of normal, dead or defective embryos not injected (WT), injected with 2 ng of *prmt8* MO or *prmt8* MO with full-length *prmt8* (FL) cRNA, catalytic inactive mutant *prmt8* (CI MT) cRNA, full-length *prmt1* cRNA, *prmt8* N-terminus with full-length *prmt1* (*prmt8+1*) cRNA, N-terminus-deleted *prmt8* (ND) cRNA, or G2A mutated *prmt8* cRNA. The embryos were examined and classified as described in Figure 3B. (B) Frequencies of different phenotypes of embryos not injected (WT), injected with 2 ng of *prmt1* MO, or *prmt1* MO with full-length (FL) *prmt1* cRNA, full-length *prmt8* cRNA, N-terminal deleted (ND) *prmt8* or *prmt8* N-terminus with full-length *prmt1* (*prmt8+1*) cRNA Injected embryos were stained with *ntl* at 10 hpf and the morphants were grouped according to the degree of shortening and widening of the notochord.

We then analyzed whether knockdown of *prmt8* may lead to reduced protein arginine methylation in the embryos. A few specific polypeptide signals detected by an ADMA-specific antibody were reduced in the *prmt8* morphants (48 hpf) (Figure S4B). Furthermore, a catalytically inactive *prmt8* cRNA could not rescue the morphants as the wildtype full-length cRNA (Figure 5A). The results suggest that Prmt8 can modify specific substrates in the embryos and the methyltransferase activity is critical for proper development.

### The functional complementation of *prmt1* and *prmt8* in early embryogenesis

As the sequences of *prmt1* and *prmt8* are highly conserved and both are expressed during early embryogenesis, we examined whether they can functionally complement each other in zebrafish early development. We used *prmt1* cRNA to rescue the *prmt8* morphants. Even though *prmt1* cRNA could not rescue the *prmt8* morphants (Figure 5A), as the major difference between Prmt1 and Prmt8 is the extra N-terminal sequence of Prmt8, we proposed that Prmt1 fused with the N-terminus of Prmt8 should function similarly as Prmt8. Indeed this cRNA rescued the morphants almost as efficiently as the *prmt8* full-length cRNA, apparently much better than the *prmt1* cRNA alone (Figure 5A).

Similarly, we examined if the *prmt1* morphants, which could be partially rescued by its own cRNA [16], can be rescued by *prmt8*. Full-length *prmt8* cRNA could barely complement the *prmt1* morphants. We postulated that the N-terminal-deleted Prmt8 should be more close to Prmt1 and thus rescued the *prmt1* morphants with the N-terminal-deleted *prmt8* cRNA. The rescue effect was close to that of *prmt1* cRNA (Figure 5B). Removal of the N-terminal 60 amino acids increased human PRMT8 activity detected *in vitro* [15]. However, the cRNA of the N-terminal deleted Prmt8 rescued the *prmt8* morphants less efficiently than the full-length *prmt8* cRNA (Figure 5A).

Myristoylation of PRMT8 was suggested to occur at the second glycine residue following the removal of the initiating methionine [11]. We thus investigated whether the residue is critical by the rescue experiment. Morphants co-injected with *prmt8* cRNA with a glycine to alanine mutation showed even more severe phenotypes than the MO2 morphants (Figure 5A).

### Defects in brain and neuronal development in the *prmt8* morphants

PRMT8 is the only brain-specific PRMT in vertebrates. As shown in the previous section, zebrafish *prmt8* was expressed in most areas of the brain at 24 hpf and *prmt8* morphants showed defects in brain development. It is likely that *prmt8* is critical for brain development. We thus stained the morphant brain with specific brain markers to illustrate the defects.

**Figure 6:**
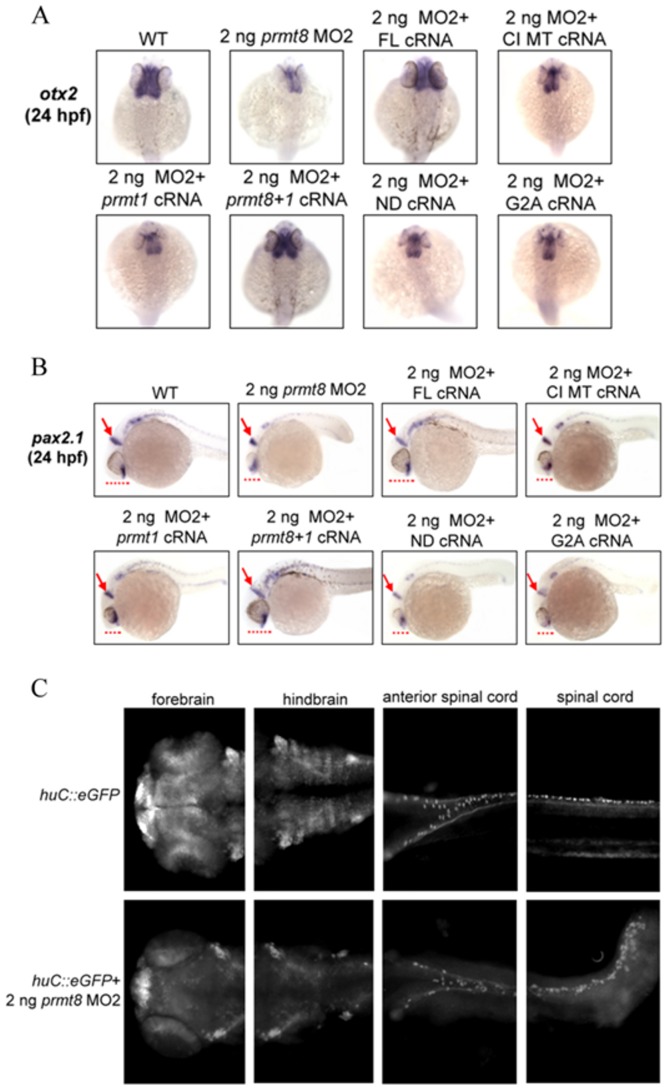
Characterization of perturbed brain phenotypes in the *prmt8* morphants. Zebrafish embryos (24 hpf) injected with 2 ng of *prmt8* MO2, or 2 ng of MO2 together with full-length (FL) *prmt8* cRNA, *prmt8* N-terminus with full-length *prmt1* (*prmt8+1*) cRNA, N-terminus-deleted (ND) *prmt8* cRNA, catalytically inactive mutant (CI MT) *prmt8* cRNA or G2A mutated *prmt8* cRNA, were stained with *otx2* (A; dorsal view with anterior to the top) or *pax2.1* (B; lateral view with anterior to the left). Arrows indicate aberrant expression patterns at the mid-hindbrain boundaries in morphant embryos. Dash lines indicate the relative length of the dorsoventral axis of the developing brain. (C) Typical embryos from the *Tg(huC::eGFP)* fish, images were taken by Leica DM2500 epifluorescence microscope with a DFC490 CCD camera.

Midbrain detected by the homeobox protein *otx2* appeared to be narrower and smaller during 24 hpf in the *prmt8* morphants and morphants rescued with *prmt1* cRNA, *prmt8* cRNA with N-terminal deletion, G2A mutation or catalytic mutation. The *otx2* expression level was reduced in the *prmt8* morphants compared with the wildtype embryos (Figure 6A). Staining of the mid-hind brain boundary with *pax2.1* also showed reduced length of the dorsoventral axis of the developing brain (Figure 6B). As the early embryonic development, full-length *prmt8* cRNA as well as cRNA expressing the N-terminus of Prmt8 fused with Prmt1 rescued the defective brain development of the *prmt*8 morphants. On the contrary, the cRNA expressing catalytic inactive, N-terminal-deleted or G2A mutated Prmt8 cannot complement. The *prmt1* cRNA could not complement either (Figure 6A, B). The results demonstrate that the methyltransferase activity as well as the N-terminus of Prmt8 are critical for the role of Prmt8 in neural patterning.

The involvement of *prmt8* in neuronal development was directly demonstrated using a transgenic *huC::eGFP* fish with fluorescent neurons. Injection of *prmt8* MO greatly decreased the HuC-positive neurons in the brain as well as in the spinal cord of the transgenic fish (Figure 6C). However, the shrunk tails may lead to the condense neurons in the tails of the 48 hpf-morphants. Not only the number of neurons decreased but also the organization of neurons was disrupted, suggesting the requirement of *prmt8* for neurogenesis as well as neural/brain patterning. Co-injection with *prmt8* cRNA brought back the fluorescent neurons. Furthermore, *prmt1* cRNA did not rescue but the cRNA expressing Prmt1 fused with the N-terminus of Prmt8 re-established the neurons in the *prmt8* morphants (data not shown).

We also stained apoptotic cells in the morphants by acridine orange, a nucleic acid intercalating dye emitting green fluorescence when bound to double-stranded DNA. In the 24-hpf MO2 morphants, concentrated fluorescent dots in the brain and somites indicate massive apoptosis in these regions. Rescue of the morphants with full-length RNA significantly released the apoptosis since much fewer acridine orange stains were detected (Figure S5A). The apoptosis was not due to off-target induction of p53 since co-injection of p53 MO did not reduce the apoptosis signals (Figure S5B).

## Discussions

In this study we use zebrafish as a vertebrate model to study the specific role of PRMT8 and the putative redundancy with its paralogue PRMT1. In contrast to other PRMTs that are widely expressed in different tissues, Prmt8 expression is limited to brain [11] and is neuron-specific [13]. During mouse development, *prmt8* transcript was exclusively expressed in brain and spinal cord at E16.5 [13]. Prmt8 protein was not detected through E13 to E17, but appeared from P0, increased thereafter and peaked at P14 [14]. Whether *prmt8* can express and function during earlier embryogenesis was not reported in these studies. In this study, we showed that *prmt8* is ubiquitously expressed from cleavage to early segmentation stages in zebrafish, similar to its paralogue *prmt1* [16], *prmt4,* and *prmt5* [25]. We further showed the requirement of *prmt8* in early development through the AMO knockdown experiments. Phenotypes as delayed epiboly and thick germ rings were observed shortly upon the injection of *prmt8* MO. Defects in epiboly and convergence/extension led to further short trunk or curly tails at later stages. Perturbed expression pattern or level of a few mesodermal, endodermal as well as ectodermal markers indicate the broad effects of *prmt8* function during early embryogenesis. The ubiquitous early expression and morphant phenotypes indicate that *prmt8* play critical roles at early embryonic stages before the development of the nervous system.

At 24 hpf, significant expression of *prmt8* was detected at eyes and brain, mid-hindbrain boundary, and also in somites. Exclusive brain expression was detected at 96 hpf. Malformed and smaller brain observed in the *prmt8* morphants were further characterized with brain specific markers. The importance of Prmt8 in neuronal development was illustrated by knockdown *prmt8* in a transgenic *Tg(huC::eGFP)* zebrafish with specific fluorescent neurons. Knockdown of *prmt8* apparently decreased the mature neurons expressing HuC. The results further confirm that *prmt8* is critical for proper neuronal development. Moreover, acridine orange staining also showed concentrated apoptotic cells in the brain and somites at 24 hpf in the *prmt8* morphants, suggesting that at least part of the loss of HuC-positive neurons was due to apoptosis. Injection of cRNA rescued the apoptosis of the morphants, further confirmed the importance of *prmt8*.

PRMT8 is a vertebrate-specific type I protein arginine methyltransferase with high sequence identity with PRMT1, the most predominant type I PRMT highly conserved in eukaryotes [2], [10]. Type I activity of zebrafish Prmt8 was determined in this study. Even though we did not expect Prmt8 to be the major type I methyltransferase, reduced arginine methylation in a few polypeptides was clearly detected in the *prmt8* morphants at 48 hpf (Figure S4). The results suggest that Prmt8 has its own substrate sets during early embryogenesis and other type I PRMTs including Prmt1 cannot complement the methylation upon *prmt8* knockdown. Furthermore, the catalytically inactive *prmt8* cannot rescue the morphants, indicating that the methyltransferase activity is essential for its physiological function.

The neuron-specific expression pattern of PRMT8 is consistent with the theme that the majority of the vertebrate-specific genes were involved in the nervous system. Although whole genome REST/NRSF target analyses have identified *prmt8* (*hrmt1l3/4*) as one of the targets [26], [27], no further attention was paid to this gene for the regulation. The NRSF/REST regulon appears to be restricted in vertebrates [26]. We thus would like to indicate that the NRSE elements should be responsible for the vertebrate-specific neuronal expression of PRMT8. The NRSF/REST protein is expressed in non-neuronal systems as a repressor to limit the expression of its target genes in neurons. However, because NRSF is expressed ubiquitously during early development in zebrafish [28], the regulation of the early ubiquitous expression of *prmt8* may involve other factors and requires further analyses.

While PRMT8 shares high sequence identity and conserved genomic structures with PRMT1, it contains an extra N-terminal sequence for about 70-90 amino acid residues in different species. This sequence is encoded by exon 1 and the 5’-half of exon 2 (Figure1A). It is likely that in vertebrate ancestors, a duplicated *prmt1* gene acquired the exons coding the N-terminus along with the NRSE/RE-1 sequence for the neuron-specificity at its 5’region. It is to note that a glutamine-rich sequence is present in the N-terminus of zebrafish but not human PRMT8. Whether this segment might have specific function for zebrafish Prmt8 requires further investigation. Moreover, a putative *prmt8*-like gene on chromosome 18 also shares identical genome structure and high amino acid sequence identity. Genome duplication events was hypothesized before the teleost radiation and recent studies further suggest that some duplications are recent lineage-specific events in zebrafish [29]. As we cannot detect mRNA expression of the gene (data not shown), consistent with no EST support, we propose that it is a non-transcribed pseudogene by duplication.

The ubiquitous expression of *prmt8* during early embryogenesis as *prmt1* suggests the putative functional redundancy at least at this stage. In the present study, we conducted knockdown-rescue experiments in the zebrafish system to analyze whether the duplicated *prmt8* and *prmt1* genes can complement each other. PRMT1 shares higher sequence homology with the N-terminus-deleted PRMT8. The cRNA encoding the N-terminal deleted but not full-length PRMT8 can complement *prmt1* morphants to a similar extent as the *prmt1* cRNA. Interestingly, we only detected limited complementation of the N-terminal deleted *prmt*8 cRNA to the *prmt*8 morphants. On the other hand, despite that *prmt1* cRNA cannot rescue the *prmt8* morphants, *prmt1* cRNA with the addition of the N-terminus of Prmt8 can complement *prmt8* morphants almost as efficiently as the *prmt8* full-length cRNA. Our results showed that *prmt1* and *prmt8 per se* can barely complement each other. However, the N-terminal modified cRNA with similar configuration of the other gene can rescue the morphants of the other gene. The results indicate that the PRMT1/PRMT8 conserved regions share similar catalytic activity but the extra N-terminus of PRMT8 appears to be the critical element determining the physiological function. Previous investigation indicated that this domain suppressed the methyltransferase activity of human PRMT8 and the proline-rich sequence in this region can interact with the SH3 domain [15]. Our results implicate that the N-terminal sequence of PRMT8 might be critical for interactions with other effectors for its specific functions. It may regulate the methyltransferase activity and recruit specific substrates. Furthermore, besides the complementation of early embryogenesis, the *prmt1* cRNA fused with the N-terminus of Prmt8 can rescue the perturbed brain development and HuC-expressing neurons of the *prmt8* knocked-down fish as the *prmt8* full-length cRNA. It can also rescue the apoptosis in the brain and somites of the *prmt8* morphants, indicating that the N-terminal region is also critical for the Prmt8 function in neuronal development and survival.

Our knockdown-rescue results suggest that *prmt8* and *prmt1* have their own specific roles in zebrafish development. We also used the knockdown-rescue system to analyze some arguments on PRMT8. PRMT8 was proposed to be membranes-bound through its N-terminal myristoylation at the second glycyl residue after the cleavage of the initiating methionine [11]. Even though the plasma membrane localization was demonstrated in HeLa cells via the expression of exotic *prmt8* [11], another investigation showed that the endogenous PRMT8 is present in the nucleus but not cell membrane, and is expressed about the size translated from the third initiator [14]. In this way, PRMT8 might be membrane-bound and more likely to participate upstream in neuronal signal transduction pathways, or it might be involved in downstream epigenetic transcription regulation as a coactivator/repressor. We prepared the cRNA with a glycine to alanine mutation at the Gly-2 position. If translation of the zebrafish *prmt8* gene is initiated at the 2^nd^ AUG (corresponds to the third initiator in mammals), there will be no myristoylation signal in the protein. If the myristoylation is not critical, we expect that this cRNA should rescue the *prmt8* morphant in a similar way as the wild-type. However, co-injection of the G2A cRNA not only cannot rescue the morphants but also led to more severe phenotypes compared with injection of *prmt8* MO alone. Whether the myristoylation is critical or this mutant cRNA might led to certain dominant negative effects requires further investigation.

In conclusion, our study provides the first illustration of early embryonic expression *prmt8* in vertebrates. The function of *prmt8* depends on its methyltransferase activity and its integrate N-terminus. The N-terminal sequence of PRMT8 might be critical for the specific interactome and substrate sets. The non-redundant roles of *prmt8* in embryonic and neuronal development with *prmt1* suggest that this vertebrate-specific gene have acquired and evolved novel functions after its duplication from the highly conserved *prmt1*.

## Supporting Information

Figure S1 **Knockdown of** ***p53*** **did not rescue the defects of** ***prmt8*** **morphant.** Zebrafish embryos injected with 2 ng of MO2 were co-injected with *p53* MO or not. Similar defects and defect rates were observed at 24 hpf in both population.
(TIF)

Figure S2 **Localization of putative NRSEs in the zebrafish** ***prmt8*** **gene.** The region displayed in this view comprises genome coordinates of the zebrafish chromosome 4 (in Jul. 2010 Zv9/danRer7 assembly) from 10,212,500 to 10,230,500 using UCSC Genome browser. The gene is annotated on the negative strand as indicated by the “>>>” symbols on the line. The GC percent in 5-base window is represented in the top track and Refseq *prmt8b* gene, non-zebra fish gene, human proteins, zebrafish mRNA in Genbank, spliced zebrafish ESTs tracks are represented in the second, 3rd, 4th, 5th and 6th lines, respectively. Comparative genomic information is represented the fish conservation by Phastcons (5 species), Fish conserved element (5 species), and Multiz Alignment and Conservation in the 7th, 8th and 9th lines, respectively.
(TIF)

Figure S3 **Validation of the inhibitory efficiency of the** ***prmt8*** **morpholino.** Embryos were injected with a vector expressing Prmt8-GFP fusion protein or the vector together with MO2 and then examined by fluorescent microscopy.
(TIF)

Figure S4 **Type I protein arginine methyltransferase activity of zebrafish Prmt8.** (A) GST-fused recombinant zebrafish Prmt8 was expressed in *Escherichia coli* and purified. *In vitro* methylation was conducted with Prmt8 and recombinant mouse fibrillarin as the methyl-accepting protein in the presence of 1.5 µCi of [methyl-^3^H]-AdoMet (60 Ci/mmol, Amersham Biotech) at 37°C for 60 min in a total volume of 15 µl in reaction buffer (50 mM sodium phosphate, pH 7.5). The samples were subjected to SDS-PAGE. The gels were then stained, treated with EN^3^HANCE (Perkin Elmer) and dried for fluorography. Control reactions with methyl-accepting protein (fibrillarin) or methyltransferase (GST-ZF8) only were conducted. (B) Reduced asymmetric dimethylarginine polypeptide signals in zebrafish *prmt8* morphants. Embryos injected with 2 ng of MO2 (MO) or not (WT) were collected at 48 hpf. Fifty microgram of embryonic extract protein was subjected to western blot analyses with an asymmetric dimethylarginine-specific antibody (ASYM24). β-actin was used as a loading control.
(TIF)

Figure S5 **Knockdown of** ***prmt8*** **leads to apoptosis in embryos.** Apoptosis analysis of zebrafish embryos at 24 hpf were conducted by acridine orange stain. (A) Embryos not injected, injected with standard AMO, injected with 2 ng *prmt8* MO2 or 2 ng *prmt8* MO2 and full-length *prmt8* cRNA are shown. (B) Embryos not injected, injected with 2 ng *prmt8* MO2, or 2 ng *prmt8* MO2 and *p53* MO are shown.
(TIF)
