# Supplementary figures and images for: The Critical Role of Protein Arginine Methyltransferase prmt8 in Zebrafish Embryonic and Neural Development Is Non-Redundant with Its Paralogue prmt1
Source: PLoS One. 2013 Mar 12;8(3):e55221. doi: 10.1371/journal.pone.0055221 (PMC3595262; doi:10.1371/journal.pone.0055221)

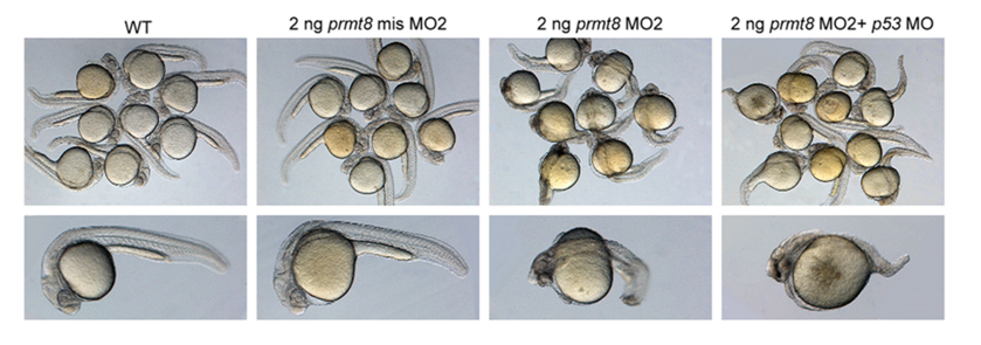

Supplement: Figure S1 — Knockdown of p53 did not rescue the defects of prmt8 morphant. Zebrafish embryos injected with 2 ng of MO2 were co-injected with p53 MO or not. Similar defects and defect rates were observed at 24 hpf in both population. (TIF) [file pone.0055221.s001.tif]

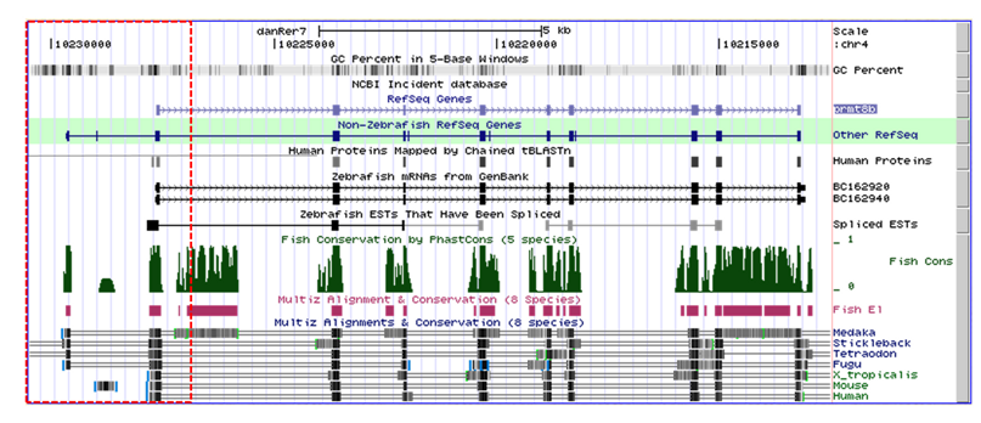

Supplement: Figure S2 — Localization of putative NRSEs in the zebrafish prmt8 gene. The region displayed in this view comprises genome coordinates of the zebrafish chromosome 4 (in Jul. 2010 Zv9/danRer7 assembly) from 10,212,500 to 10,230,500 using UCSC Genome browser. The gene is annotated on the negative strand as indicated by the “>>>” symbols on the line. The GC percent in 5-base window is represented in the top track and Refseq prmt8b gene, non-zebra fish gene, human proteins, zebrafish mRNA in Genbank, spliced zebrafish ESTs tracks are represented in the second, 3rd, 4th, 5th and 6th lines, respectively. Comparative genomic information is represented the fish conservation by Phastcons (5 species), Fish conserved element (5 species), and Multiz Alignment and Conservation in the 7th, 8th and 9th lines, respectively. (TIF) [file pone.0055221.s002.tif]

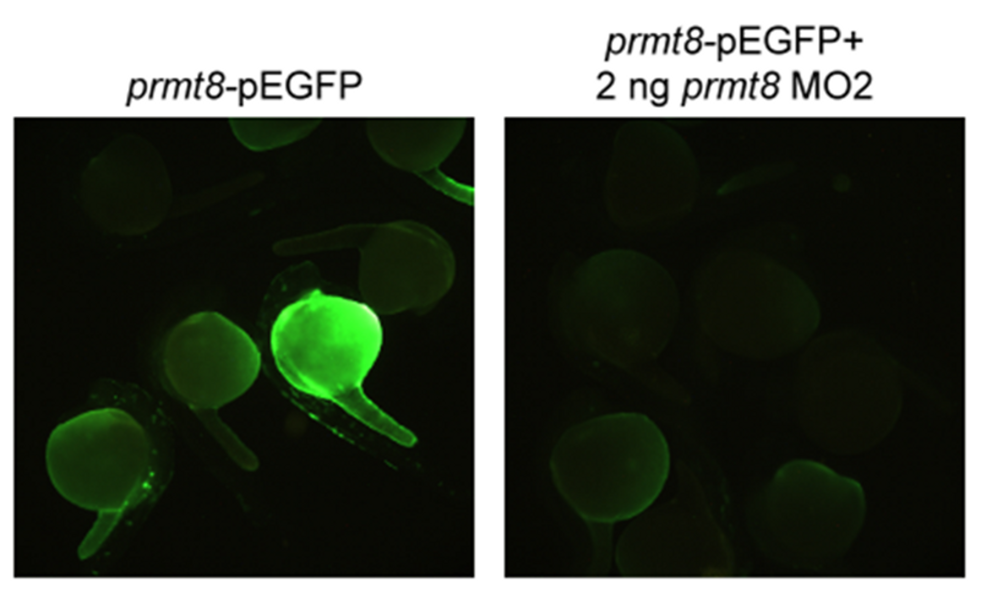

Supplement: Figure S3 — Validation of the inhibitory efficiency of the prmt8 morpholino. Embryos were injected with a vector expressing Prmt8-GFP fusion protein or the vector together with MO2 and then examined by fluorescent microscopy. (TIF) [file pone.0055221.s003.tif]

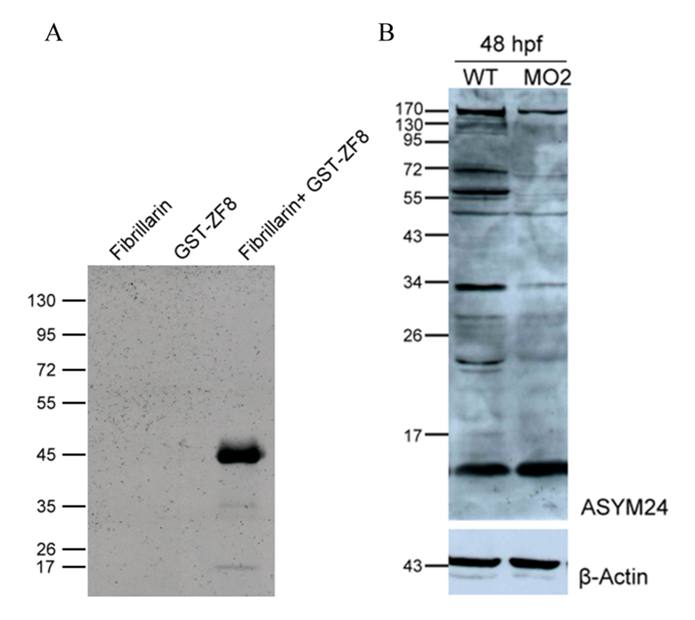

Supplement: Figure S4 — Type I protein arginine methyltransferase activity of zebrafish Prmt8. (A) GST-fused recombinant zebrafish Prmt8 was expressed in Escherichia coli and purified. In vitro methylation was conducted with Prmt8 and recombinant mouse fibrillarin as the methyl-accepting protein in the presence of 1.5 µCi of [methyl-3H]-AdoMet (60 Ci/mmol, Amersham Biotech) at 37°C for 60 min in a total volume of 15 µl in reaction buffer (50 mM sodium phosphate, pH 7.5). The samples were subjected to SDS-PAGE. The gels were then stained, treated with EN3HANCE (Perkin Elmer) and dried for fluorography. Control reactions with methyl-accepting protein (fibrillarin) or methyltransferase (GST-ZF8) only were conducted. (B) Reduced asymmetric dimethylarginine polypeptide signals in zebrafish prmt8 morphants. Embryos injected with 2 ng of MO2 (MO) or not (WT) were collected at 48 hpf. Fifty microgram of embryonic extract protein was subjected to western blot analyses with an asymmetric dimethylarginine-specific antibody (ASYM24). β-actin was used as a loading control. (TIF) [file pone.0055221.s004.tif]

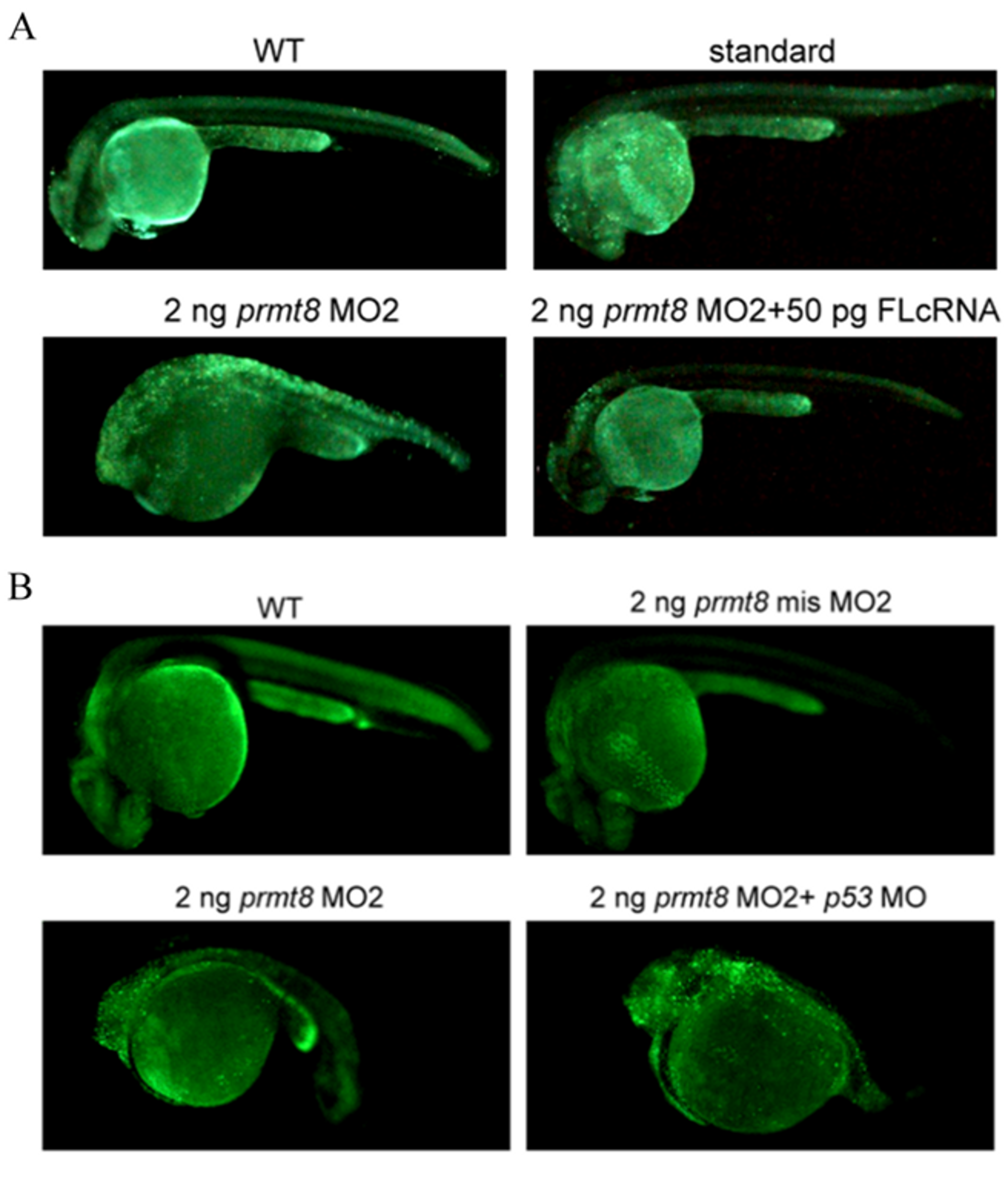

Supplement: Figure S5 — Knockdown of prmt8 leads to apoptosis in embryos. Apoptosis analysis of zebrafish embryos at 24 hpf were conducted by acridine orange stain. (A) Embryos not injected, injected with standard AMO, injected with 2 ng prmt8 MO2 or 2 ng prmt8 MO2 and full-length prmt8 cRNA are shown. (B) Embryos not injected, injected with 2 ng prmt8 MO2, or 2 ng prmt8 MO2 and p53 MO are shown. (TIF) [file pone.0055221.s005.tif]
